# Comparison of three imaging and navigation systems regarding accuracy of pedicle screw placement in a sawbone model

**DOI:** 10.1038/s41598-022-16709-y

**Published:** 2022-07-19

**Authors:** Nils Beisemann, Jula Gierse, Eric Mandelka, Frank Hassel, Paul A. Grützner, Jochen Franke, Sven Y. Vetter

**Affiliations:** 1 Research Group Medical Imaging and Navigation in Trauma and Orthopedic Surgery (MINTOS), Berufsgenossenschaftliche Unfallklinik (BG Trauma Center) Ludwigshafen, Ludwig-Guttmann-Strasse 13, 67071 Ludwigshafen, Germany; 2 Department of Spine Surgery, Loretto Hospital, Mercystrasse 6, 79100 Freiburg im Breisgau, Germany

**Keywords:** Fracture repair, Medical imaging, Bone, Skeleton, Trauma

## Abstract

3D-navigated pedicle screw placement is increasingly performed as the accuracy has been shown to be considerably higher compared to fluoroscopy-guidance. While different imaging and navigation devices can be used, there are few studies comparing these under similar conditions. Thus, the objective of this study was to compare the accuracy of two combinations most used in the literature for spinal navigation and a recently approved combination of imaging device and navigation system. With each combination of imaging system and navigation interface, 160 navigated screws were placed percutaneously in spine levels T11-S1 in ten artificial spine models. 470 screws were included in the final evaluation. Two blinded observers classified screw placement according to the Gertzbein Robbins grading system. Grades A and B were considered acceptable and Grades C-E unacceptable. Weighted kappa was used to calculate reliability between the observers. Mean accuracy was 94.9% (149/157) for iCT/Curve, 97.5% (154/158) for C-arm CBCT/Pulse and 89.0% for CBCT/StealthStation (138/155). The differences between the different combinations were not statistically significant except for the comparison of C-arm CBCT/Pulse and CBCT/StealthStation (p = 0.003). Relevant perforations of the medial pedicle wall were only seen in the CBCT group. Weighted interrater reliability was found to be 0.896 for iCT, 0.424 for C-arm CBCT and 0.709 for CBCT. Under quasi-identical conditions, higher screw accuracy was achieved with the combinations iCT/Curve and C-arm CBCT/Pulse compared with CBCT/StealthStation. However, the exact reasons for the difference in accuracy remain unclear. Weighted interrater reliability for Gertzbein Robbins grading was moderate for C-arm CBCT, substantial for CBCT and almost perfect for iCT.

## Introduction

Pedicle screw (PS) placement has been considered a standard procedure in spine surgery for many years and is widely used for a variety of indications^1,2^. Due to the close anatomical relation of the pedicle and sensitive structures, PS placement carries the risk of a number of complications, including neurological, vascular or dural injury following perforation of the pedicle wall^3–5^. Another challenge is the interindividual differences in pedicle morphology, which require individual surgical planning before and possible adjustments during surgery^6,7^. The accuracy of freehand PS placement is generally considered acceptable. However, the accuracy rates available in the literature differ substantially^8^.

The aforementioned reasons and the trend towards minimally invasive techniques have led to the increasing use of intraoperative 2D and 3D imaging and navigation in PS placement^9–12^. The available literature contains several studies including systematic reviews and meta-analyses showing a significantly lower risk for screw malposition in 3D navigated PS placement compared to both freehand and fluoroscopy-controlled techniques^13–17^.

For 3D navigation, an intraoperative 3D imaging device as well as a compatible navigation system is needed. For intraoperative 3D imaging, either Cone Beam CT (CBCT) or intraoperative CT (iCT) imaging can be used and both result in the intraoperative generation of a 3D dataset, yet, besides the technical aspect, these methods differ in terms of field of view (FOV) and image quality^18–22^. While spinal navigation solutions are available from different manufacturers, integrated imaging and navigation systems from the same manufacturer are most commonly used in combination^23^.

While CBCT has been performed for about two decades and especially the O-arm (Medtronic, Dublin, Ireland) in combination with StealthStation navigation (Medtronic, Dublin, Ireland) seems to be widely used in spine surgery, iCT-based navigation has been increasingly applied in recent years since the introduction of the mobile iCT Airo (Brainlab, Munich, Germany) and Curve navigation (Brainlab, Munich, Germany)^23^.

The iCT, in general, has the advantage of a higher image resolution and a larger FOV, whereas with CBCT the radiation dose for the patient is lower and the use of the device more feasible^24–26^. Furthermore, there are differences between the available CBCT systems, like gantry size and form (O-arm vs. C-arm) as well as the detector technology (image-intensifier vs. flat panel technology) integrated^27^.

While numerous clinical studies have investigated navigated PS placement, yet, the conditions that these studies are performed under vary greatly, which makes it difficult to compare the clinical use of different imaging devices and navigation systems^15,28,29^. Studies conducted under standardised experimental conditions are lacking in the literature. Therefore, the purpose of this study was to compare the ‘gold standard’ iCT with the most widely used CBCT and a novel system for C-arm CBCT-based navigation and to evaluate the accuracy of each system for thoracolumbar PS placement in an experimental setting.

## Methods

**Figure 1:**
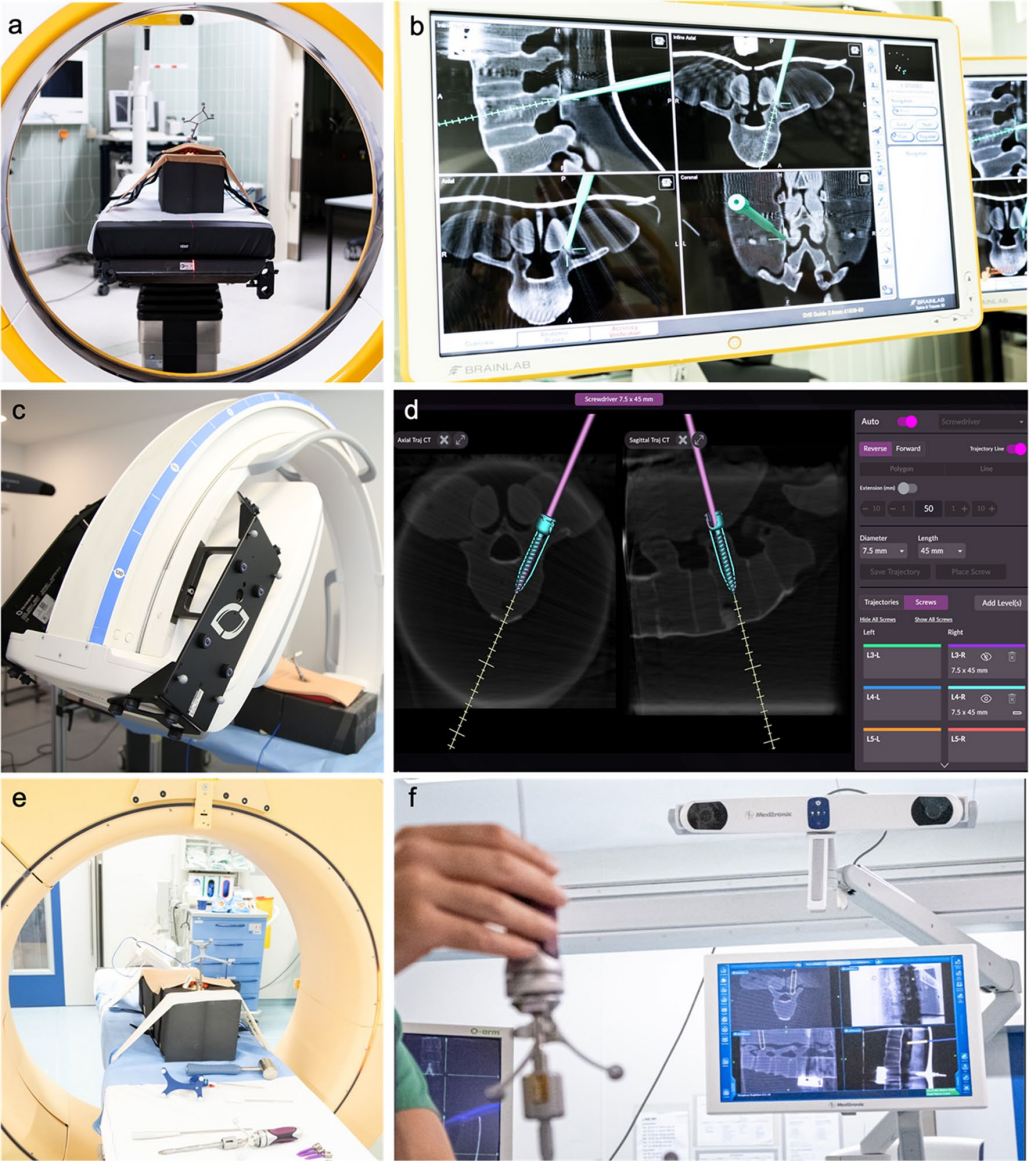
The images devices and respective navigation interfaces used in the study: iCT (**a**) with curve navigation (**b**), C-arm CBCT (**c**) with pulse navigation (**d**) and CBCT (**e**) with StealthStation navigation (**f**).

In this experimental study, dorsal instrumentation of screws was performed on 30 identical radiopaque artificial spine models (Synbone, Zizers, Switzerland) using three different systems for imaging and navigation (Fig. 1): A mobile intraoperative CT (iCT; Airo, Brainlab, Munich, Germany) with Curve Image-Guided Surgery navigation (Version Spine&Trauma 3D 2.6, Brainlab, Munich, Germany), a mobile flat-panel C-arm (C-arm CBCT; Cios Spin, Siemens Healthcare, Forchheim, Germany) coupled with the novel navigation software of the Pulse platform (Version 4.0.1, NuVasive, San Diego, California, USA) and the 1^st^ generation O-arm system (CBCT; Medtronic, Dublin, Ireland) with its designated StealthStation navigation (Synergy Spine S7, Version 2.1.0, Medtronic, Dublin, Ireland).

The artificial spine models included the spinal segments T10 to the sacral bone. With each system, bilateral pedicle screws (Reline MAS, NuVasive, San Diego, California, USA) were placed in spine levels T11 to S1 in ten artificial bone models, resulting in 160 screws per system and 480 screws in total. PS placement was performed 50:50 by two senior physicians. The first surgeon is an experienced spine surgeon with several years of experience with navigated PS placement, while the second one has had training in spine surgery equivalent to a senior resident. PS placement was performed on three separate days, each approximately three months apart, with placement of all screws using a combination of imaging system and navigation solution on one day at a time. The trials with iCT/Curve were performed first, followed by those with CBCT/StealthStation and finally C-arm CBCT/Pulse.

Prior to PS placement, the appropriate screw length and diameter for the spine model were determined for each pedicle on a 3D scan. For T11 and T12, 6.5 × 40 mm screws were selected. For L1 to L4, 7.5 × 45 mm screws were used. For L5 7.5 × 40 mm screws and for S1 7.5 × 35 mm screws were chosen.

The spine bed, including the spine model with muscles and synthetic skin, was placed on the operating table in the prone position. For all imaging systems, the procedure was similar. First, after skin incision and preparation of the soft tissue, the referencing array was mounted on the spinous process of L2.

PS placement was performed minimally invasive. After determination of the desired point and angle of entry with the navigation pointer, skin incision and preparation of the soft tissue to the entry point were performed. The navigated pointer was used to locate the point of entry again under verification of the trajectory in three planes using the respective navigation system. A navigated drill guide was used in all cases to achieve a maximum of accuracy in contrast to tapping or using navigated Jamshidi needles. After drilling under permanent control of trajectory and drilling depth, a K-wire was inserted. The previously selected screw was placed into the pedicle over the wire with the navigated screwdriver, again under permanent control of trajectory and drilling depth. Finally, the K-wire was removed. This procedure was repeated for every screw. With each combination of imaging device and navigation solution, surgeons placed screws on both sides in five sawbone models in a row. As a result, the same number of screws was placed on both sides by each surgeon.

**Figure 2:**
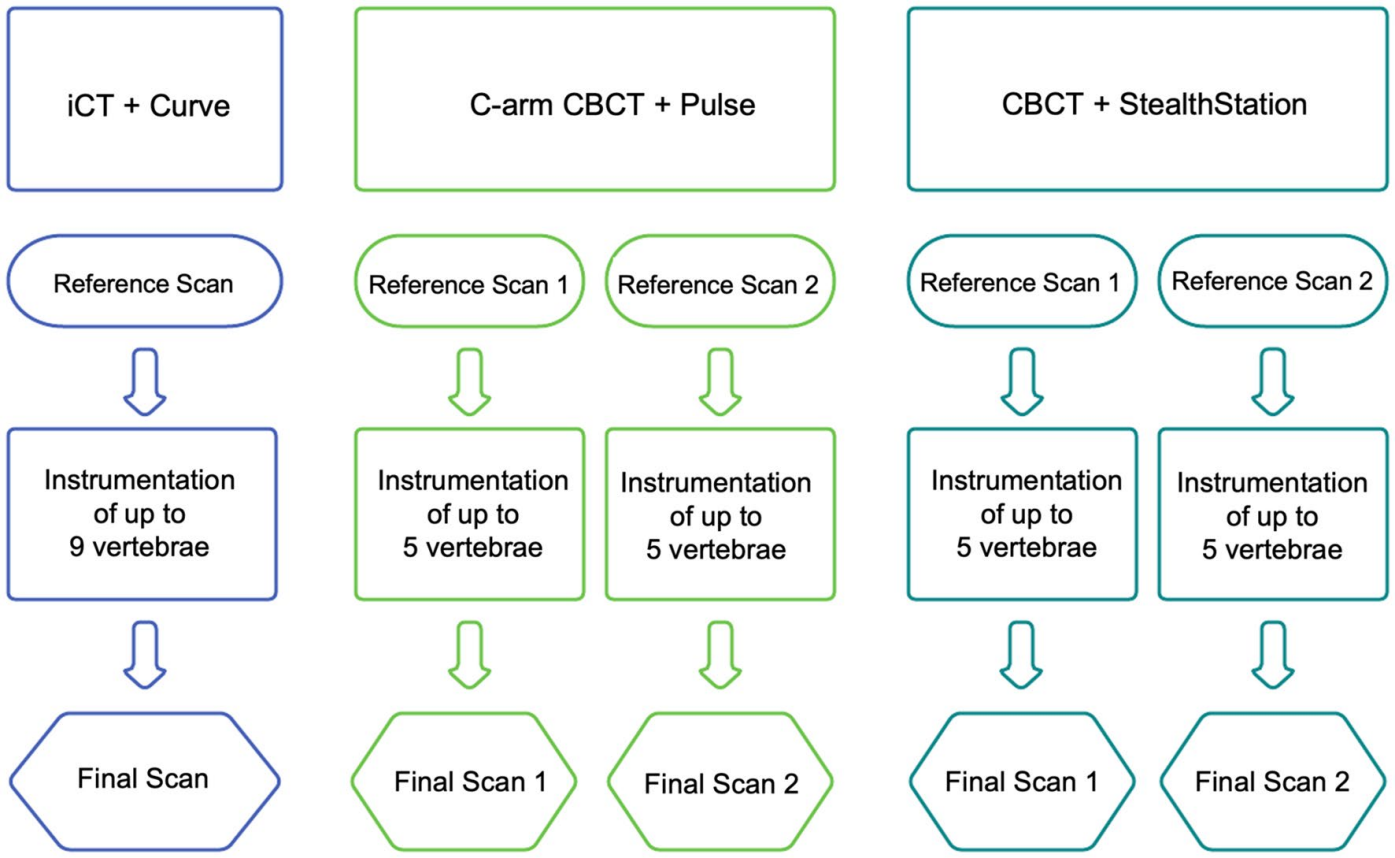
Study design. For the C-arm CBCT/Pulse and the CBCT/StealthStation groups two scans had to be performed for referencing as well as evaluation due to the smaller field of view.

Using the iCT/Curve for navigation, all 16 screws were placed continuously (Fig. 2). After screw placement, one final scan for evaluation was performed. In the C-arm CBCT/Pulse and CBCT/StealthStation groups, a second 3D referencing scan had to be obtained after placing screws in spinal levels T11 to L3 due to the smaller FOV. After that, the screws were placed in the remaining pedicles, followed by two scans to evaluate PS accuracy. For all devices, 3D imaging was performed with the standard radiation protocols (iCT: adult lumbar spine, patient weight 70 kg; C-arm CBCT: standard quality, 200 images in 30 s; CBCT: patient size medium).

**Figure 3:**
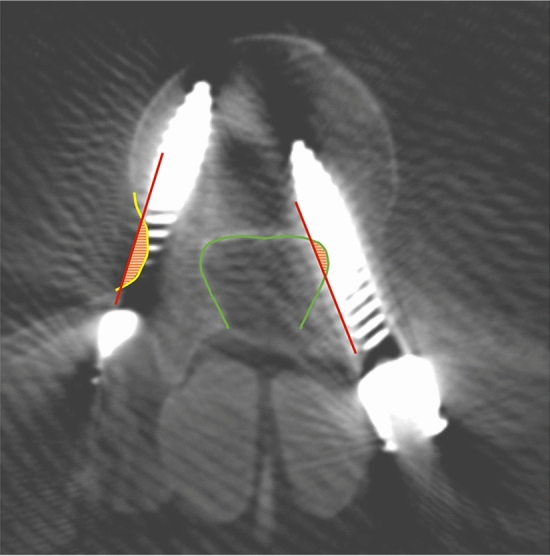
Illustrative case of screw assessment. Perforation of the medial pedicle wall by the left screw, perforation of the lateral pedicle wall by the right screw.

All acquired image datasets of the different imaging systems were subsequently analysed by two independent observers using a DICOM viewer (Fig. 3). The image data was pseudononymized to allow blinding of the two observers. The distance of each screw to the medial and lateral cortex was measured and classified according to GRS^30^. As common in the literature, pedicle perforations ≥ 2 mm (Grade C-E) were considered as potentially clinically relevant and therefore deemed unacceptable^20,31^.

Kappa (κ) was used to calculate interrater reliability of GRS between the two observers. In order to account for close matches of the two assessments, Kappa was weighted (κ_weighted_). Consequently, a rating of A by Observer 1 and B by Observer 2 is weighted higher than a rating of A by Observer 1 and D by Observer 2. Interpretation of kappa was made according to Landis and Koch (< 0.00 no agreement, 0.00–0.20 slight agreement, 0.21–0.40 fair agreement, 0.41–0.60 moderate agreement, 0.61–0.80 substantial agreement, 0.81–1.00 almost perfect agreement)^32^. Fisher’s exact test was used to compare screw accuracy between the two surgeons.

The data tabulated in Excel (Microsoft Excel 2019, version 16.38) was analysed using ANOVA tests and two-tailed Fisher’s exact test in JMP (Version 14.2.0, SAS, Cary, USA). Kolmogorov–Smirnov-Test was used to test for normality distribution. Figures were created using Prism 8 (Graphpad Software, San Diego, USA). The significance level was set at p < 0.05.

## Results

In total, 470 of the planned 480 screws could be placed. Ten screws (iCT/Curve 3, C-arm CBCT/Pulse 2, CBCT/StealthStation 5) could not be placed because of material defects of the spine models that caused the pedicle to break when the screw was placed, making accurate screw placement impossible. All screws placed could be assessed according to GRS by the two observers (Obs1/Obs2).

PS placement accuracy for the three different combinations of imaging device and navigation system as assessed by the two observers is presented in Table 1.

**Table 1:** Pedicle perforations in the different groups.

	iCT/curve		C-arm CBCT/Pulse				CBCT/StealthStation	
	Obs1	Obs2	Obs1		Obs2		Obs1	Obs2
**Overall perforations**	29/157 (18.5%)	36/157 (22.9%)	28/158 (17.7%)		24/158 (18.4%)		60/155 (38.7%)	58/155 (37.4%)
Of which ≥ 2 mm (GRS C-E)	7 (4.5%)	9 (5.7%)	3 (1.9%)		5 (3.2%)		18 (11.6%)	16 (10.3%)
Of which medial	0 (0.0%)	0 (0.0%)	0 (0.0%)		0 (0.0%)		2 (11.1%)	2 (12.5%)
Of which lateral	7 (100.0%)	9 (100.0%)	3 (100.0%)		5 (100.0%)		16 (88.9%)	14 (87.5%)
PS placement accuracy	150/157 (95.5%)	148/157 (94.3%)	155/158 (98.1%)		153/158 (96.8%)		137/155 (88.4%)	139/155 (89.5%)

*Obs1* Observer 1, *Obs2* Observer2, *GRS C-E* Gertzbein Robbins Grade C–E.

PS placement using iCT and Curve navigation revealed relevant perforations in 4.5% (Obs 1) and 5.7% (Obs2) of screws. In the group of C-arm CBCT/Pulse navigated screws, perforations corresponding to Grades C-E according to GRS were assessed in 1.9% (Obs1) and 3.2% (Obs2) of screws, respectively, with all screws perforating the lateral pedicle wall. In the CBCT/StealthStation group, pedicle perforations ≥ 2 mm were assessed in 11.6% (Obs1) and 10.3% (Obs2) of screws, respectively. Of all the screws to which this applied, two perforated the medial pedicle wall.

**Figure 4:**
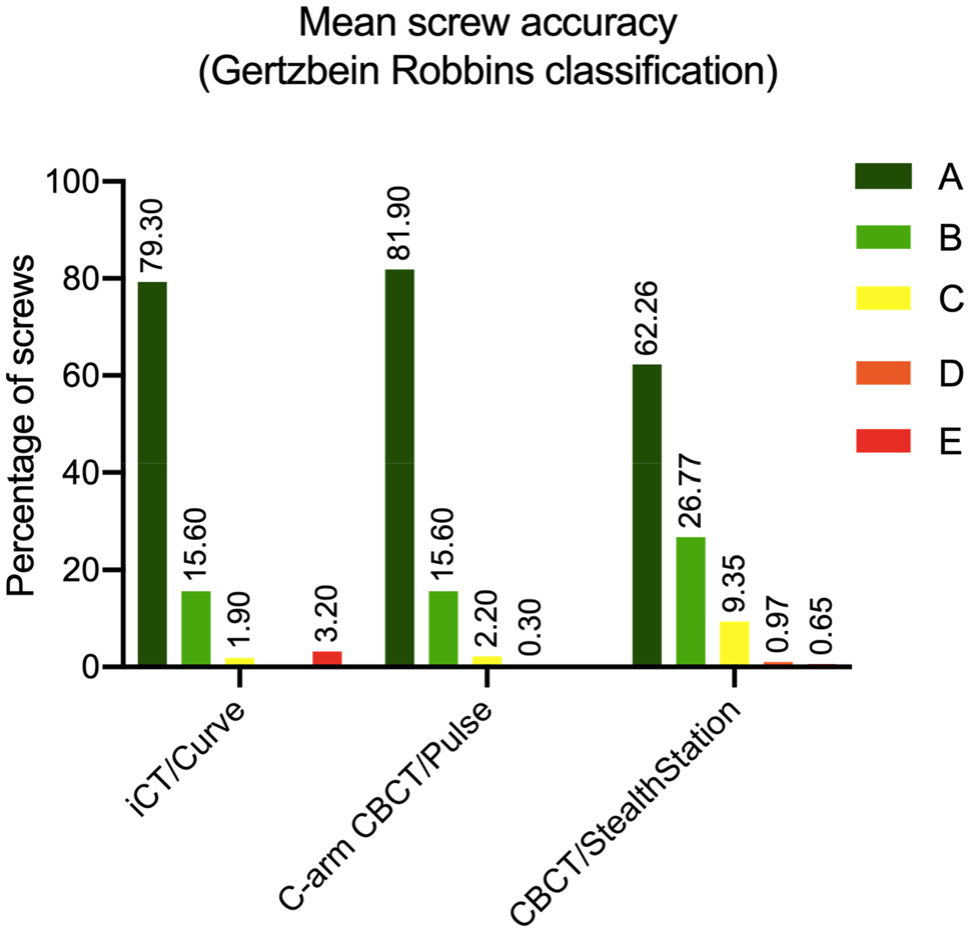
Mean accuracy of screw placement for the two observers according to GRS.

The mean percentage of screws classified as Grade A-E according to Gertzbein and Robbins is illustrated in Fig. 4. The difference between the groups regarding the rate of relevant pedicle perforations was significant (p = 0.02, ANOVA). Fisher’s exact test showed a significant difference between C-arm CBCT/Pulse and CBCT/StealthStation (p = 0.003). No significant differences were found for the comparison of iCT/Curve and the CBCT/StealthStation (p = 0.06) as well as iCT/Curve and C-arm CBCT/Pulse (p = 0.26).

**Figure 5:**
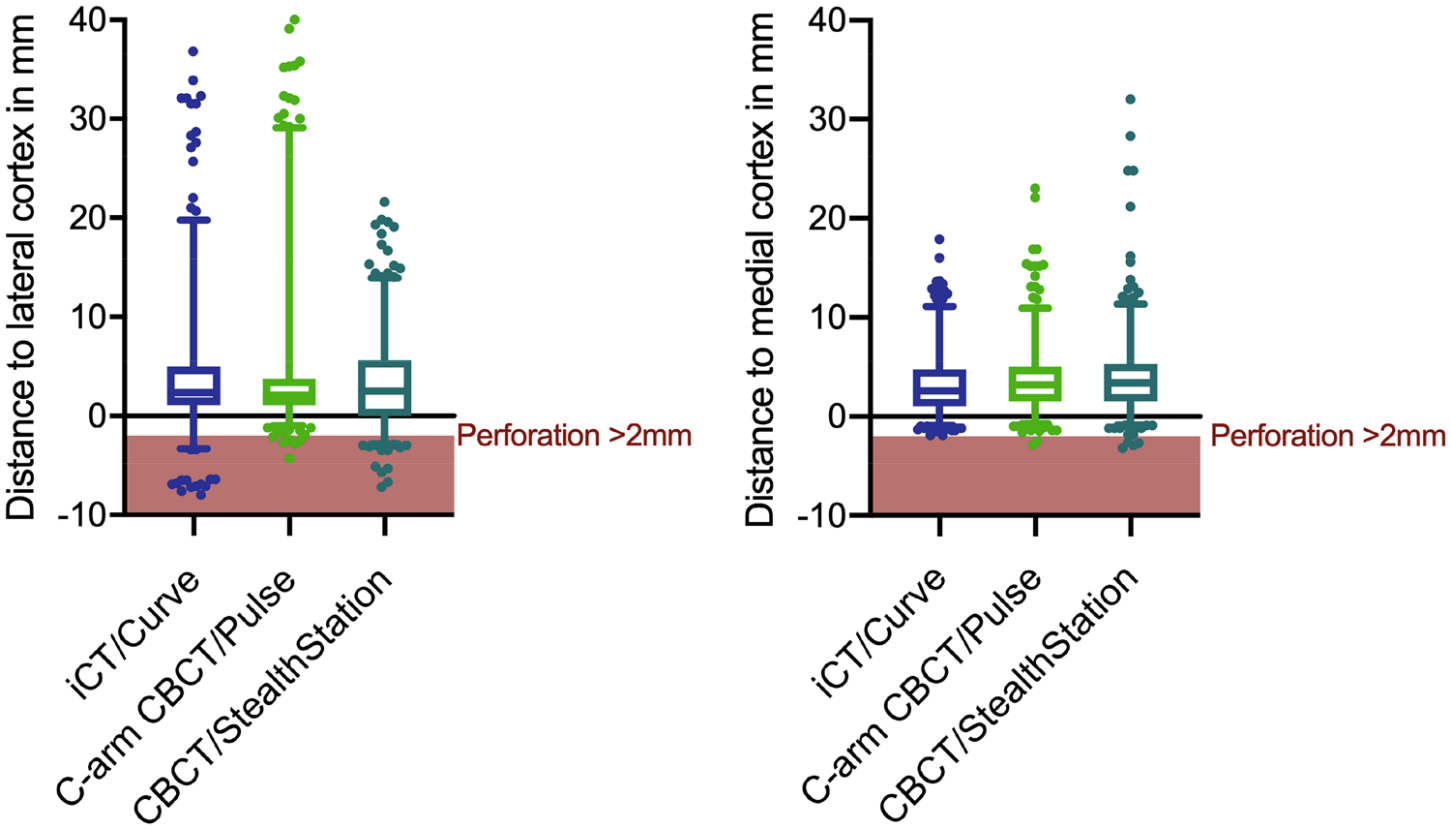
Mean distance to lateral and medial pedicle cortex with 95% confidence interval as assessed by the observers dependent on the combination of imaging system and navigation system used. The measurements from both observers are displayed resulting in two data points for each screw. The screws in the area marked in red represent the clinically relevant perforations.

Overall, regardless of the combination used, 26 of 28 (Obs1) and 28 of 30 (Obs2) perforations assessed by the observers involved the lateral pedicle wall. Perforation of the medial wall ≥ 2 mm occurred only in the CBCT/StealthStation group (Fig. 5).

**Figure 6:**
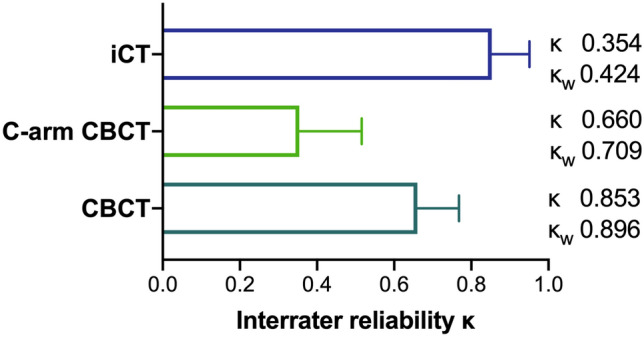
Interrater reliability κ and κ_weighted_ (κ_w_) of GRS assessment for the different imaging devices investigated.

For 5.1% (iCT), 19.6% (C-arm CBCT) and 18.1% (CBCT) of the screws placed, a different Gertzbein Robbins grade was assigned by the two observers. Differences in the assessment of a screw as clinically acceptable (grades A/B) or not (grades C-E) were seen in 1.3% (iCT), 3.8% (C-arm CBCT) and 8.3% (CBCT) of the screws, respectively. Interrater reliability κ was moderate (0.41–0.60) for C-arm CBCT/Pulse (0.424), substantial (0.61–0.80) for CBCT/StealthStation (0.709) and almost perfect (0.81–1.00) for iCT/Curve (0.896; Fig. 6).

No significant differences were found for the two surgeons regarding screw accuracy as assessed by the two observers.

## Discussion

**Figure 7:**
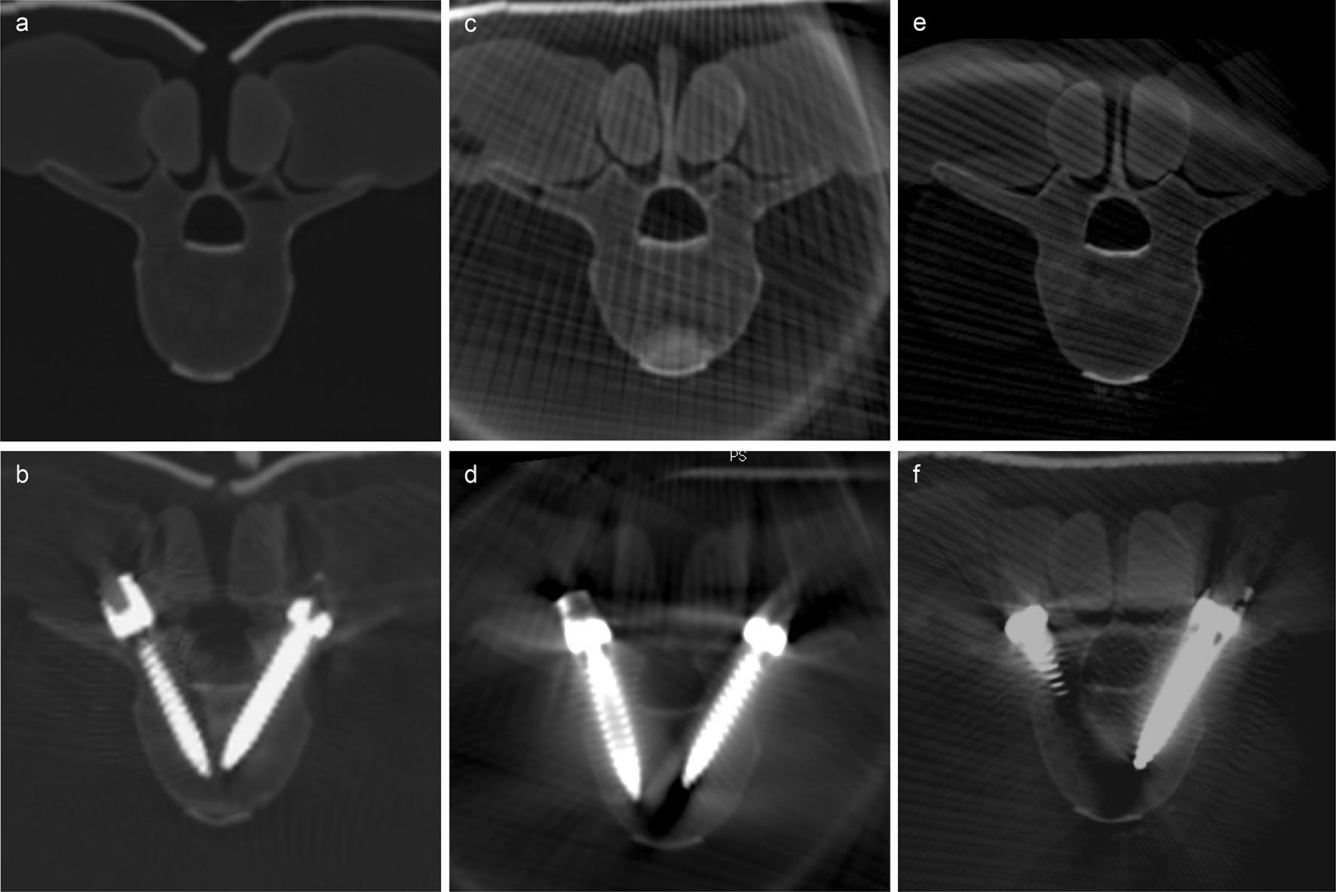
Visualization of pedicles before (**a,c,e**) and after screw placement (**b,d,f**) in the lumbar spine (L3) in intraoperative imaging. (**a/b**) iCT, (**c/d**) C-arm CBCT, (**e/f**) CBCT.

The aim of this study was to compare three different imaging and navigation systems regarding the accuracy of PS placement (Fig. 7). In contrast to the already existing clinical studies, this was carried out under experimental, quasi-identical conditions. The accuracy was investigated by placing a total of 470 screws in 30 artificial spine models, with ten models being assessed for each imaging and navigation system. The accuracy of every screw placed was subsequently evaluated by two observers based on the acquired 3D imaging using the GRS. Screws with a pedicle perforation of 2 mm or more were considered unacceptable.

From a methodological point of view, the procedures in the different groups differ mainly insofar as only one scan is required for referencing and evaluation with the iCT due to the larger FOV of up to 51cmx100cm, whereas two scans each are required for referencing and evaluation with both the C-arm CBCT and the CBCT. For surgeries with instrumentation of more than five segments, the larger FOV of the iCT can be advantageous, especially for intraoperative workflow. On the other hand, it should be noted that the accuracy of navigation applications may decrease with increasing distance from the reference base, and so performing two scans with a smaller FOV may be advantageous for accuracy^33^. Furthermore, for surgeries with iCT that also require conventional 2D fluoroscopy, an additional device may be needed^27^. In this regard, C-arm CBCT and CBCT appear to be the more feasible solutions with greater flexibility of use.

The use of iCT with Curve navigation and C-arm CBCT with Pulse navigation showed comparable mean results with 94.9% and 97.5% respectively in contrast to 89.0% for CBCT and StealthStation navigation.

It should be noted that while for the iCT/Curve and C-arm CBCT/Pulse groups no relevant perforation of the medial pedicle cortex was measured, two medial cortex perforations were noticed for the CBCT/StealthStation trial. Medial perforations have a higher risk of causing clinically relevant complications and should therefore be considered as critical. Still, not all perforations measured cause clinical symptoms. This is especially true for lateral perforations, so avoiding such a screw position was not a primary goal for the surgeons in our study. Furthermore, screws with the largest diameter possible for the related pedicle were chosen in this study, favoring the perforation probability in the porous spine models. The selected screw size has already been described by Burström et al. as a reason for varying accuracy of PS placement^34^. Since, regardless of the combination of imaging device and navigation system used, medial perforations occurred in only 0.4% of all screws, PS placement was considered safe with all imaging and navigation systems.

While 5.1% (iCT), 19.6% (C-arm CBCT) and 18.1% (CBCT) of the screws were assigned a different Gertzbein Robbins grade by the two observers. The highest clinical relevance of the differences was seen for assessment in CBCT with 8.3% of screws being assessed differently regarding the clinical relevance of the pedicle perforation. Interrater reliability was almost perfect for iCT/Curve and substantial for CBCT/StealthStation. For C-arm CBCT/Pulse interrater reliability was moderate only, because there was a comparatively high number of screws with a divergence in clinically irrelevant grades A and B between the observers.

The interrater reliability for GRS reported by different studies in the literature ranges from 0.53 for three observers and 0.45 for four observers^35,36^. Comparing our results to the literature proved to be difficult as no studies were found reporting interrater agreement for two observers only. Burström et al. reported an absolute interrater agreement between three observers of 72.9% for CT and 63.1% for CBCT, however, interrater reliability calculated as kappa was 0.48 for CT and 0.63 for CBCT which led the authors to draw the conclusion that CBCT is reliable to rule out pedicle perforation intraoperatively, making postoperative CT unnecessary^37^.

The results obtained regarding accuracy using iCT/Curve and C-arm CBCT/Pulse are similar to data published in the literature regarding percutaneous navigated PS placement.

Tkatschenko et al. also compared the accuracy of iCT- and C-arm CBCT guided Curve navigated PS placement in 75 patients. For PS placement, a similar technique was used as in our study (percutaneous, using a guide wire etc.). However, instead of a mobile C-arm CBCT, the authors used a stationary ceiling-mounted robotic C-arm system (Artis Zeego, Siemens Healthcare, Forchheim, Germany) and the same navigation software (Curve, Brainlab, Feldkirchen, Germany)^38^. To the best of our knowledge, no studies on the navigation of transpedicular screws for the mobile C-arm CBCT used in our study are available to date. For the Pulse navigation system (Nuvasive, San Diego, California, USA) presented in this study, the results are also the first to be published.

Similar to our approach, Tkatschenko et al. also placed screws in the thoracolumbar to sacral vertebrae and screw position was also evaluated according to the GRS. An accuracy of 95.5% for iCT and 95.8% C-arm CBCT was reported with no significant difference between the two systems. Furthermore, Hecht et al. achieved an accuracy rate of 96.0% in the thoracolumbar spine using the iCT and Curve with the same criteria for evaluation, and the same device and navigation system as used in our study^39^.

Therefore, the literature seems to confirm our results for iCT guided Curve navigated PS placement, while for the CBCT/StealthStation group our findings differ substantially from previously published results: According to Van de Kelft et. al who also investigated pedicle screw accuracy using the same CBCT and navigation system that were used in this study, 97.5% of 1922 screws were placed correctly. Yet, they used a different method to evaluate screw accuracy with a cut-off of 50% of the screw diameter for lateral perforations. Medial perforations were considered unacceptable in any case^40^.

A similar accuracy (97.7%) is also reported by Vardiman et al. in the lumbosacral spine using GRS for evaluation, although the referencing scans were performed not only with the CBCT used in our study but in some cases with a conventional computed tomography scanner. In addition, the placement was performed using a robot-guided navigation solution, which could explain the higher accuracy^41^.

In their trial, Farah et al. compared navigated PS placement with CBCT/StealthStation and iCT/Curve. The authors reached an accuracy rate of 90.8% and 92.2%, respectively. However, in contrast to our methods, screws were placed in the thoracic spine only and, besides GRS, accuracy was also evaluated based on the Heary classification^24^. Different methods of PS assessment have been shown to massively influence reporting of screw accuracy. In their meta-analysis, Kosmopoulos et al. report that only 50% of the included studies even specified in detail how the evaluation was performed. Considering only those studies with a detailed description of the procedure, the accuracy rate decreased from 91.3 to 86.7%. Furthermore, in view of 35 different evaluation methods in the studies considered, the authors conclude that a uniform method for assessing the accuracy of PS placement is necessary^42^.

Comparability is further limited by the fact that all the aforementioned results reflect clinical data collected during PS placement in patients, whereas our investigations were conducted in an experimental setting using artificial spine models. The spine models used in this study are challenging when it comes to accurate pedicle screw placement, as there is no difference between cortical and cancellous bone which results in screws not running along the cortex but rather perforating it. This goes unnoticed as there is no haptical feedback in terms of the typical resistance when perforating the cortex^33^.

In addition to the reasons explained in relation to the model used, there are other reasons that could explain the differences in screw accuracy observed in this study. In this regard, causes within the scope of screw placement itself and the assessment of accuracy must be differentiated.

First, image quality is of great importance for navigated screw placement and is mainly influenced by the amount of soft tissue surrounding the spine. As sawbone models with almost no soft tissue were used in this study, image quality was not relevant for screw placement itself. For screw evaluation, on the other hand, image quality may—depending on the imaging device—be highly influenced by metal artefacts. Comparative studies on image quality in posterior fixation have been performed in a specimen setting by Keil et al. and, more recently, Foster et al. who both investigated imaging devices also used in this study^27,43^. Their results indicate that the assessment of screw accuracy obtained in the present study may be due to the misplacement rate being overestimated as a consequence of the amount of artefacts surrounding the screw, which may also be indicated in Fig. 7. A closer look at our results regarding the interrater agreement reveals a substantial difference when comparing C-arm CBCT and CBCT in terms of absolute agreement of GRS and agreement in terms of clinically relevant classification as A/B or C-E. While CBCT showed complete agreement for 81.9% of screws, C-arm CBCT did so in 80.4% of screws. In contrast, the interrater agreement for the assessment of clinical relevance was lower with CBCT (91.6%) compared to C-arm CBCT (96.2%). This may be caused by a potentially more metal artefacts, resulting in appearing to be larger in diameter in CBCT and thus the degree might be overestimated.

Second, the navigation system used is of course a potential influencing factor. Although no technical accuracy was collected as a comparison between planned and actual trajectory in the context of this clinical study, it is unlikely that the observed differences are due to technical inaccuracies, as accuracy is extensively checked before FDA and CE approval, respectively.

Thus, the third and presumably decisive factor is the execution of screw placement. On one hand, this depends on the skill and experience of the surgeon^28^. With regard to experience, although the more experienced spine surgeon had several years of experience with iCT navigated pedicle screw placement, the presence of a learning curve within the series of experiments with the two systems he was unfamiliar with cannot be entirely ruled out. For the second investigator with less experience, the existence of a potential learning curve seems to be more relevant. While this bias was minimized between the different combinations by choosing a time interval of approximately three months between each series of experiments, the lack of experience in the beginning could explain the rate of high-grade perforations that occurred in the iCT/Curve series that was performed first^44^. All screws assessed as grade E were placed by this surgeon in the smallest pedicles in terms of diameter (2 × T11, 2 × 12, 1 × L1).

Beyond the direct influence of surgeon skill and experience, different factors have been identified by other authors that influence the quality of execution. One factor is the mobility of the spine leading to undetected movements of the instrumented vertebra in relation to the vertebra the patient array is attached to. This has already been described by Miller et al. and Frisk et al. and might go unnoticed during screw placement. Another related reason is unintentional or unnoticed contact with the reference array placed close to the surgical field^33,45^. Furthermore, from our own experience, inaccuracies in screw placement might also be the result of inadequate screwdriver-screw-connection, especially in polyaxial screws. The fact that even in robot-assisted studies with a specified screw trajectory, an accuracy of 100% cannot always be achieved can be seen as an indication that the surgeon can minimize the abovementioned causes by regularly checking accuracy and trying not to distort the anatomy, yet, they cannot be completely prevented^41,46,47^.

However, the most important limitation of this study is that despite its experimental study design, it was not possible to identify the reasons for the differences in inaccuracy. Nevertheless, this is the first study in which the screw accuracy is compared using three combinations of imaging device and navigation system under quasi-identical conditions which is only possible to a very limited extent, if at all.

In addition, the clinical relevance is enhanced by the fact that we investigated two commonly used combinations and a third novel combination, that has not yet been reported on in the literature.

To differentiate the impact of the imaging devices including the image quality from the effect of the navigation system used, a combination of every imaging device with every navigation system is needed. However, as discussed above, other factors would still need to be taken into account which further increases the requirements of the study design.

For the reasons discussed above, the use of sawbone models is a further limitation of the study. Because of the very limited clinical relevance as a result of the lack of soft tissue around the sawbone models, image quality was not assessed in this study. Accordingly, the comparability with clinical application could be significantly increased by the use of human specimens, yet, a study with a relevant number of screws placed to compare screw accuracy seems hardly feasible due to the extreme requirements, both financially and in terms of resources. This may be the reason why no representative studies of this kind have been performed so far.

In our study, accuracy was evaluated using GRS, with considerable variation in the cut-off values available in the literature to distinguish relevant from irrelevant screw misplacements^25,42,48–50^. Nevertheless, GRS is the most common classification for assessing PS placement in the available literature^16,51^. Therefore, in the absence of better alternatives, the classification was applied in this study. For future work, the definition of uniform criteria for the assessment would be desirable, ideally based on outcome-relevant factors. Therefore, in addition to the experimental studies needed to identify the influence of several factors on screw accuracy, clinical studies should aim to investigate the potential impact of increased accuracy of PS placement on patient outcome.

Under quasi-identical conditions, we found differences in screw accuracy for the combinations iCT/Curve and C-arm CBCT/Pulse compared with CBCT/StealthStation, yet they were not statistically significant except for the comparison of C-arm CBCT/Pulse and CBCT/StealthStation. However, the exact reasons for the difference in accuracy remain unclear. Weighted interrater reliability for Gertzbein Robbins grading was moderate for C-arm CBCT, substantial for CBCT and almost perfect for iCT.

## Data Availability

All data and statistics are available on reasonable request from the corresponding author.
